# Fluconazole–malonic acid (1/1)

**DOI:** 10.1107/S1600536813003656

**Published:** 2013-02-13

**Authors:** Jože Kastelic, Danijel Kikelj, Ivan Leban, Nina Lah

**Affiliations:** a Krka d.d., Šmarješka cesta 6, 8501 Novo mesto, Slovenia; b University of Ljubljana, Faculty of Pharmacy, Aškerčeva 7, 1000 Ljubljana, Slovenia; c University of Ljubljana, Faculty of Chemistry and Chemical Technology, Aškerčeva 5, 1000 Ljubljana, Slovenia

## Abstract

Co-crystallizaton of the anti­fungal drug fluconazole [2-(2,4-difluoro­phen­yl)-1,3-bis­(1*H*-1,2,4-triazol-1-yl)propan-2-ol] with malonic acid in acetonitrile solution resulted in the formation of the title 1:1 co-crystal, C_13_H_12_F_2_N_6_O·C_3_H_4_O_4_. The geometry around the central fluconazole atom is distorted tetrahedral. The dihedral angles between the triazole rings and the fluorinated phenyl ring are 30.64 (7) and 61.91 (5)°. In the crystal, the basic packing motif may be envisioned as a cyclic aggregate formed of two fluconazole mol­ecules linked by two malonic acid mol­ecules through O—H⋯N and O—H⋯O hydrogen bonds. Such aggregates are further connected into (001) layers by further O—H⋯N hydrogen bonds. The structure also features weak non-classical C—H⋯O and C—H⋯N inter­actions.

## Related literature

For general aspects of pharmaceutical co-crystals, see, for example: Brittain *et al.* (2012*a* ▶,*b* ▶). For known fluconazole co-crystals, see: Kastelic *et al.* (2010 ▶, 2011 ▶). For regulatory classification of pharmaceutical co-crystals, see: US Food and Drug Administration (2011 ▶).
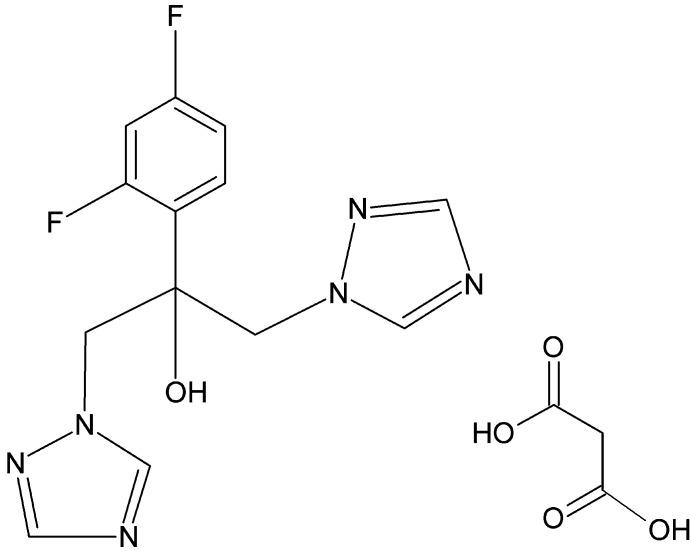


## Experimental

### 

#### Crystal data

C_13_H_12_F_2_N_6_O·C_3_H_4_O_4_
*M* *_r_* = 410.35
Orthorhombic, 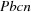

*a* = 14.7196 (2) Å
*b* = 8.4891 (1) Å
*c* = 28.1096 (4) Å
*V* = 3512.47 (8) Å^3^
*Z* = 8
Mo *K*α radiation
μ = 0.13 mm^−1^
*T* = 150 K
0.18 × 0.18 × 0.12 mm

#### Data collection

Nonius Kappa CCD diffractometer
7522 measured reflections
4012 independent reflections
3069 reflections with *I* > 2σ(*I*)
*R*_int_ = 0.024

#### Refinement

*R*[*F* ^2^ > 2σ(*F* ^2^)] = 0.038
*wR*(*F* ^2^) = 0.100
*S* = 1.03
4012 reflections
274 parameters
H atoms treated by a mixture of independent and constrained refinement
Δρ_max_ = 0.32 e Å^−3^
Δρ_min_ = −0.29 e Å^−3^

### 

Data collection: *COLLECT* (Nonius, 1998 ▶); cell refinement: *DENZO* and *SCALEPACK* (Otwinowski & Minor, 1997 ▶); data reduction: *DENZO* and *SCALEPACK*; program(s) used to solve structure: *SHELXS97* (Sheldrick, 2008 ▶); program(s) used to refine structure: *SHELXL97* (Sheldrick, 2008 ▶); molecular graphics: *ORTEP-3 for Windows* (Farrugia, 2012 ▶); software used to prepare material for publication: *SHELXL97* and *PLATON* (Spek, 2009 ▶).

## Supplementary Material

Crystal structure: contains datablock(s) global, I. DOI: 10.1107/S1600536813003656/gk2552sup1.cif

Structure factors: contains datablock(s) I. DOI: 10.1107/S1600536813003656/gk2552Isup2.hkl

Supplementary material file. DOI: 10.1107/S1600536813003656/gk2552Isup3.cml

Additional supplementary materials:  crystallographic information; 3D view; checkCIF report

## Figures and Tables

**Table 1:** Hydrogen-bond geometry (Å, °)

*D*—H⋯*A*	*D*—H	H⋯*A*	*D*⋯*A*	*D*—H⋯*A*
O1—H1⋯O11*M*	0.89 (2)	1.94 (2)	2.8057 (14)	166.2 (18)
O12*M*—H11⋯N14^i^	0.88 (2)	1.86 (2)	2.6830 (17)	156 (2)
O21*M*—H12⋯N24^ii^	0.88 (3)	1.89 (3)	2.7606 (17)	171 (2)
C6—H6⋯N14^i^	0.93	2.61	3.484 (2)	156
C12*M*—H12*B*⋯O11*M* ^iii^	0.97	2.44	3.3880 (18)	165
C13—H13⋯O12*M* ^iv^	0.93	2.54	3.3144 (19)	141
C25—H25⋯O11*M*	0.93	2.40	3.2018 (18)	144
C25—H25⋯O22*M* ^iii^	0.93	2.37	3.0032 (19)	125

Symmetry codes: (i) 
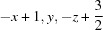
; (ii) 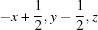; (iii) 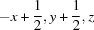; (iv) 
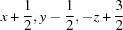
.

**Fig. 1.**
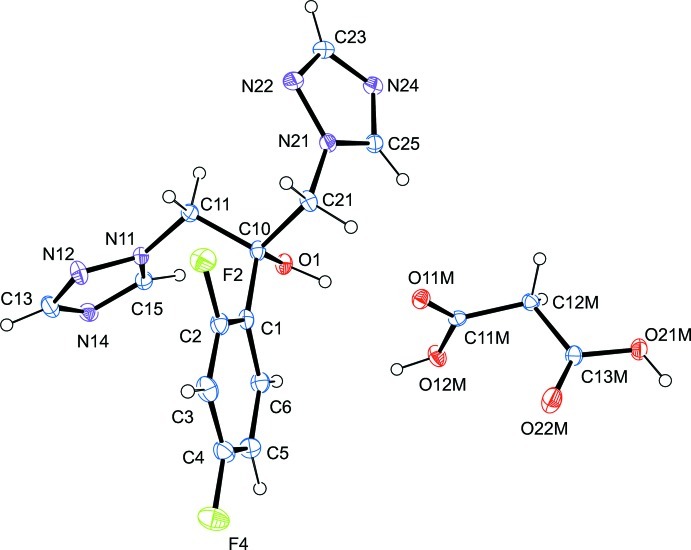
The asymmetric unit of the title compound, showing the atom-numbering scheme. Displacement ellipsoids are drawn at the 30% probability level.

**Fig. 2.**
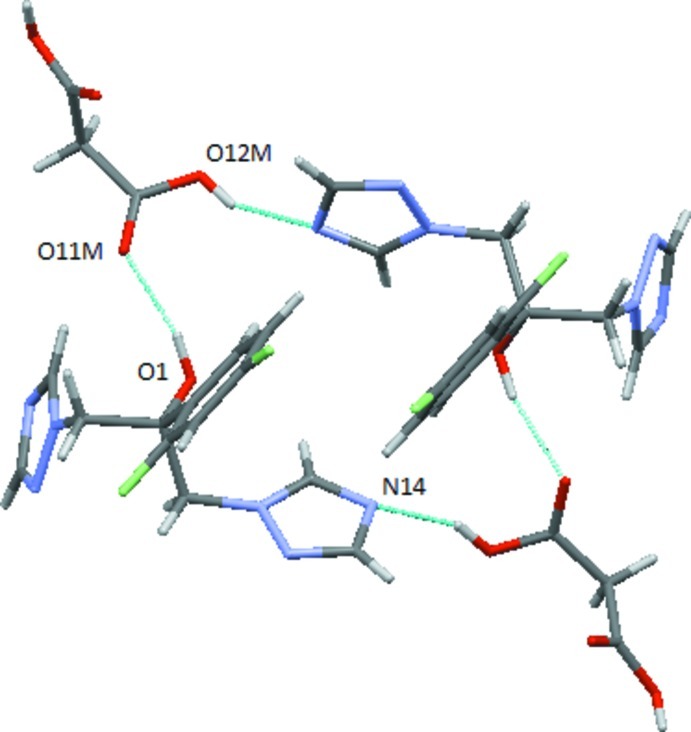
Part of the crystal structure showing the formation of a cyclic hydrogen bonded heterotetramer built from two fluconazole and two malonic acid molecules (projection down the b axis). Symmetry code for generation of the tetramer: -x+1, y, -z+1.5.

**Fig. 3.**
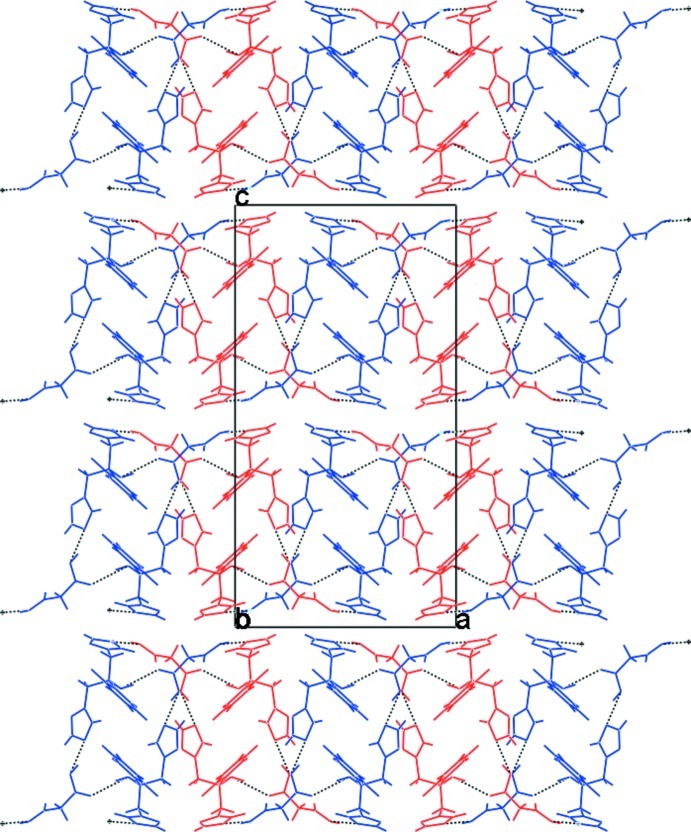
Two-dimensional (001) layers viewed down the b axis. The tetrameric buliding units are alternately coloured red and blue to emphasize their linkage. Hydrogen bonds are indicated as dashed lines.

## supplementary crystallographic information

### Comment

There has been an intense interest in the preparation and characterization of pharmaceutical cocrystals which is evident from the increasing number of research publications on this topic in the past decade (Brittain, 2012*a*,*b* and references therein). Pharmaceutical cocrystals present a sub-set of multicomponent crystal solid forms of the active pharmaceutical ingredient (API) with the ability to modulate API's physicochemical properties and to provide intellectual property implications. Until now no cocrystal drug substances have received regulatory approval yet. The relevance of cocrystals in drug formulation research and development has been confirmed with the publication of new U.S. Food and Drug Administration's (FDA) draft guidance for the regulatory classification of pharmaceutical cocrystals in December 2011 (US Food and Drug Administration, 2011).

Fluconazole is a wide spectrum triazole antifungal agent which is only slightly soluble in water. It is a weak base (p*K*_a_ value of 1.76 for its conjugated acid). Therefore, the formation of a salt as a mean to improve solubility properties, could only be expected with very strong acids. On the other hand, cocrystallization offers possibilities to influence the solubility with numerous coformers. Along these lines, we have focused our research on the preparation of new fluconazole cocrystals. The results of our systematic cocrystallization screening are the cocrystals of fluconazole with three dicarboxylic acids, namely maleic, glutaric and fumaric (Kastelic *et al.*, 2010) and a cocrystal with salicylic acid (Kastelic *et al.*, 2011). As a continuation of our work we present here the crystal structure of a 1:1 cocrystal of fluconazole with malonic acid.

The asymmetric unit consists of one fluconazole and one malonic acid molecule, both in their neutral forms (Fig. 1). Both crystal formers can act as donors and/or acceptors in hydrogen bonding. As expected, the packing arrangement of the two molecules in the crystal is governed by hydrogen-bond interactions. A basic packing motif may be envisioned as a ring in which two fluconazole molecules (related by a two fold axis; symmetry code: +1-x, y, +1.5-z) are bridged by two malonic acid molecules by hydrogen bond interactions including fluconazole OH group as a donor to carboxylic O atom of malonic acid (for hydrgen bond geometry see Table 1). Additionally, the same carboxylic group acts as a donor to one of the triazole N atoms of the fluconazole moiety (Fig. 2). Such rings are further connected into two-dimensional layers through the interaction of the second carboxylic group of malonic acid with another fluconazole triazole nitrogen atom (N24) of the adjacent building unit . The layers are oriented perpendicular to the *z* axis (Fig. 3). Additionally, the structure is stabilized by non-classical H-bond interactions (for details see Table 1).

The packing in the title compound does not resemble the structures of other known fluconazole cocrystals with carboxylic acids where the formation of zigzag coloumns or sheets were observed. The generation of a flat-layered structure provides the possibilities for the improved mechanical properties relevant to the tablet formulation. Their investigations are underway.

### Experimental

Fluconazole was obtained from Krka d.d., Novo mesto, malonic acid was obtained from Merck and both were used without further purification. Equimolar amounts of fluconazole (100 mg, 0.33 mmol) and malonic acid (34.3 mg, 0.33 mmol) were dissolved in 3 ml of acetonitrile by heating at 50°C. The clear solution was slowly cooled to ambient temperature and solvent was then allowed to evaporate to form a transparent film from which crystals grew in 72 h. Crystals suitable for single-crystal X-ray diffraction analysis were prepared by dissolving fluconazole (100.0 mg, 0.33 mmol) and malonic acid (34.3 mg, 0.33 mmol) in 3 ml of acetonitrile by heating at 50°C. Seeds from screening experiment described above were added to clear solution. The mixture was slowly cooled to ambient temperature and allowed to evaporate slowly until the crystals of suitable size and quality appeared.

### Refinement

All H atoms were initially found in a Fourier-difference map, but they were repositioned to their calculated positions and were refined using a riding model. Aromatic H atoms were permitted to ride with C—H = 0.93 Å and *U*_eq_(H) = 1.2*U*_iso_(C). H atoms of the CH_2_ group were constrained with C—H = 0.97 Å and *U*_eq_(H)=1.2*U*_iso_(C). H atoms of hydroxyl groups involved in the formation of hydrogen bonds were freely refined.

### Crystal data

**Table:**

C_13_H_12_F_2_N_6_O·C_3_H_4_O_4_	*D*_x_ = 1.552 Mg m^−^^3^
*M**_r_* = 410.35	Mo *K*α radiation, λ = 0.71073 Å
Orthorhombic, *P**b**c**n*	Cell parameters from 4524 reflections
*a* = 14.7196 (2) Å	θ = 1.0–27.5°
*b* = 8.4891 (1) Å	µ = 0.13 mm^−^^1^
*c* = 28.1096 (4) Å	*T* = 150 K
*V* = 3512.47 (8) Å^3^	Hexagonal plates, colourless
*Z* = 8	0.18 × 0.18 × 0.12 mm
*F*(000) = 1696	

### Data collection

**Table:**

Nonius Kappa CCD diffractometer	3069 reflections with *I* > 2σ(*I*)
Radiation source: fine-focus sealed tube	*R*_int_ = 0.024
Graphite monochromator	θ_max_ = 27.5°, θ_min_ = 2.0°
ω–scans at κ=55°	*h* = −19→19
7522 measured reflections	*k* = −11→11
4012 independent reflections	*l* = −36→36

### Refinement

**Table:**

Refinement on *F*^2^	Primary atom site location: structure-invariant direct methods
Least-squares matrix: full	Secondary atom site location: difference Fourier map
*R*[*F*^2^ > 2σ(*F*^2^)] = 0.038	Hydrogen site location: inferred from neighbouring sites
*wR*(*F*^2^) = 0.100	H atoms treated by a mixture of independent and constrained refinement
*S* = 1.03	*w* = 1/[σ^2^(*F*_o_^2^) + (0.0504*P*)^2^ + 1.0478*P*] where *P* = (*F*_o_^2^ + 2*F*_c_^2^)/3
4012 reflections	(Δ/σ)_max_ = 0.001
274 parameters	Δρ_max_ = 0.32 e Å^−^^3^
0 restraints	Δρ_min_ = −0.29 e Å^−^^3^

### Special details

**Table:**

Geometry. All s.u.'s (except the s.u. in the dihedral angle between two l.s. planes) are estimated using the full covariance matrix. The cell s.u.'s are taken into account individually in the estimation of s.u.'s in distances, angles and torsion angles; correlations between s.u.'s in cell parameters are only used when they are defined by crystal symmetry. An approximate (isotropic) treatment of cell s.u.'s is used for estimating s.u.'s involving l.s. planes.
Refinement. Refinement of *F*^2^ against ALL reflections. The weighted *R*-factor *wR* and goodness of fit *S* are based on *F*^2^, conventional *R*-factors *R* are based on *F*, with *F* set to zero for negative *F*^2^. The threshold expression of *F*^2^ > σ(*F*^2^) is used only for calculating *R*-factors(gt) *etc*. and is not relevant to the choice of reflections for refinement. *R*-factors based on *F*^2^ are statistically about twice as large as those based on *F*, and *R*- factors based on ALL data will be even larger.

### Fractional atomic coordinates and isotropic or equivalent isotropic displacement parameters (Å^2^)

**Table:**

	*x*	*y*	*z*	*U*_iso_*/*U*_eq_	
C1	0.54177 (9)	0.13534 (17)	0.64550 (5)	0.0206 (3)	
C2	0.58200 (10)	−0.00574 (18)	0.63211 (5)	0.0244 (3)	
C3	0.55741 (11)	−0.15059 (19)	0.64974 (6)	0.0293 (3)	
H3	0.5863	−0.2425	0.6400	0.035*	
C4	0.48774 (11)	−0.15328 (19)	0.68268 (6)	0.0288 (3)	
C5	0.44513 (11)	−0.01972 (19)	0.69784 (6)	0.0286 (3)	
H5	0.3987	−0.0248	0.7202	0.034*	
C6	0.47258 (10)	0.12392 (18)	0.67916 (5)	0.0241 (3)	
H6	0.4439	0.2154	0.6894	0.029*	
F2	0.65063 (6)	−0.00225 (11)	0.59954 (3)	0.0341 (2)	
F4	0.46114 (7)	−0.29475 (11)	0.70033 (4)	0.0425 (3)	
C10	0.57056 (9)	0.29636 (17)	0.62593 (5)	0.0200 (3)	
O1	0.51652 (7)	0.41828 (12)	0.64549 (3)	0.0222 (2)	
C21	0.56338 (10)	0.29942 (17)	0.57093 (5)	0.0230 (3)	
H21A	0.6091	0.2302	0.5575	0.028*	
H21B	0.5042	0.2604	0.5614	0.028*	
N21	0.57605 (8)	0.45798 (15)	0.55235 (4)	0.0225 (3)	
N22	0.65894 (9)	0.51379 (18)	0.53827 (5)	0.0319 (3)	
C23	0.64032 (11)	0.6622 (2)	0.52823 (6)	0.0322 (4)	
H23	0.6843	0.7322	0.5174	0.039*	
N24	0.55240 (8)	0.70550 (15)	0.53487 (4)	0.0262 (3)	
C25	0.51475 (10)	0.57304 (18)	0.55025 (5)	0.0233 (3)	
H25	0.4539	0.5620	0.5585	0.028*	
C11	0.66769 (9)	0.33814 (19)	0.64058 (5)	0.0224 (3)	
H11A	0.7095	0.2650	0.6256	0.027*	
H11B	0.6820	0.4431	0.6292	0.027*	
N11	0.68076 (8)	0.33275 (14)	0.69205 (4)	0.0206 (3)	
C15	0.64684 (10)	0.42420 (18)	0.72607 (5)	0.0231 (3)	
H15	0.6054	0.5053	0.7212	0.028*	
N14	0.68090 (9)	0.38273 (15)	0.76801 (4)	0.0260 (3)	
C13	0.73709 (11)	0.26218 (19)	0.75655 (5)	0.0292 (3)	
H13	0.7714	0.2088	0.7792	0.035*	
N12	0.73963 (9)	0.22619 (15)	0.71109 (4)	0.0288 (3)	
O11M	0.34684 (6)	0.39993 (12)	0.60035 (4)	0.0233 (2)	
O12M	0.25373 (8)	0.51564 (13)	0.65244 (4)	0.0285 (2)	
C11M	0.27804 (9)	0.47413 (16)	0.60930 (5)	0.0195 (3)	
C12M	0.21064 (9)	0.52498 (17)	0.57209 (5)	0.0209 (3)	
H12A	0.2418	0.5411	0.5421	0.025*	
H12B	0.1833	0.6242	0.5815	0.025*	
C13M	0.13749 (10)	0.40240 (17)	0.56589 (5)	0.0219 (3)	
O21M	0.06834 (7)	0.45375 (13)	0.54002 (4)	0.0286 (3)	
O22M	0.14215 (8)	0.27195 (13)	0.58274 (5)	0.0389 (3)	
H1	0.4594 (14)	0.409 (2)	0.6360 (7)	0.039 (5)*	
H11	0.2895 (15)	0.474 (3)	0.6739 (8)	0.050 (6)*	
H12	0.0268 (17)	0.380 (3)	0.5357 (9)	0.068 (8)*	

### Atomic displacement parameters (Å^2^)

**Table:**

	*U*^11^	*U*^22^	*U*^33^	*U*^12^	*U*^13^	*U*^23^
C1	0.0193 (7)	0.0243 (7)	0.0182 (6)	0.0026 (6)	−0.0046 (5)	−0.0010 (6)
C2	0.0215 (7)	0.0309 (8)	0.0208 (7)	0.0057 (6)	−0.0003 (6)	−0.0017 (6)
C3	0.0352 (8)	0.0239 (8)	0.0288 (8)	0.0076 (7)	−0.0042 (7)	−0.0020 (6)
C4	0.0347 (8)	0.0235 (8)	0.0281 (8)	−0.0023 (7)	−0.0050 (7)	0.0044 (6)
C5	0.0267 (8)	0.0319 (9)	0.0273 (8)	−0.0005 (7)	0.0019 (6)	0.0018 (6)
C6	0.0231 (7)	0.0241 (8)	0.0250 (7)	0.0042 (6)	−0.0001 (6)	−0.0018 (6)
F2	0.0322 (5)	0.0348 (5)	0.0351 (5)	0.0114 (4)	0.0111 (4)	0.0001 (4)
F4	0.0563 (6)	0.0244 (5)	0.0468 (6)	−0.0023 (5)	0.0060 (5)	0.0071 (4)
C10	0.0182 (6)	0.0231 (7)	0.0188 (7)	0.0048 (6)	−0.0018 (5)	−0.0028 (6)
O1	0.0202 (5)	0.0232 (5)	0.0233 (5)	0.0050 (4)	−0.0017 (4)	−0.0039 (4)
C21	0.0264 (7)	0.0240 (8)	0.0185 (7)	0.0038 (6)	−0.0034 (6)	−0.0002 (6)
N21	0.0206 (6)	0.0281 (7)	0.0188 (6)	0.0021 (5)	−0.0007 (5)	0.0014 (5)
N22	0.0226 (6)	0.0449 (9)	0.0282 (7)	0.0032 (6)	0.0054 (5)	0.0101 (6)
C23	0.0271 (8)	0.0390 (10)	0.0306 (8)	−0.0031 (7)	0.0039 (7)	0.0110 (7)
N24	0.0267 (6)	0.0296 (7)	0.0223 (6)	0.0001 (6)	0.0016 (5)	0.0028 (5)
C25	0.0215 (7)	0.0269 (8)	0.0215 (7)	0.0018 (6)	−0.0006 (6)	0.0007 (6)
C11	0.0202 (7)	0.0291 (8)	0.0180 (7)	0.0003 (6)	0.0002 (5)	0.0014 (6)
N11	0.0186 (5)	0.0232 (6)	0.0201 (6)	−0.0005 (5)	−0.0033 (5)	0.0020 (5)
C15	0.0226 (7)	0.0237 (8)	0.0230 (7)	−0.0001 (6)	−0.0024 (6)	0.0001 (6)
N14	0.0293 (7)	0.0272 (7)	0.0214 (6)	−0.0056 (5)	−0.0025 (5)	0.0015 (5)
C13	0.0334 (8)	0.0286 (8)	0.0255 (8)	0.0013 (7)	−0.0073 (6)	0.0054 (6)
N12	0.0309 (7)	0.0297 (7)	0.0260 (6)	0.0079 (6)	−0.0067 (5)	0.0028 (5)
O11M	0.0208 (5)	0.0226 (5)	0.0265 (5)	0.0019 (4)	−0.0007 (4)	−0.0007 (4)
O12M	0.0301 (5)	0.0361 (6)	0.0193 (5)	0.0079 (5)	−0.0008 (5)	−0.0002 (5)
C11M	0.0203 (7)	0.0157 (7)	0.0227 (7)	−0.0030 (6)	0.0015 (5)	0.0008 (5)
C12M	0.0217 (7)	0.0199 (7)	0.0211 (7)	0.0010 (6)	−0.0001 (5)	0.0018 (5)
C13M	0.0214 (7)	0.0213 (8)	0.0229 (7)	0.0025 (6)	−0.0023 (6)	−0.0019 (6)
O21M	0.0254 (6)	0.0267 (6)	0.0338 (6)	−0.0024 (5)	−0.0108 (5)	0.0061 (5)
O22M	0.0306 (6)	0.0216 (6)	0.0646 (8)	−0.0022 (5)	−0.0169 (6)	0.0094 (6)

### Geometric parameters (Å, º)

**Table:**

C1—C2	1.388 (2)	C23—H23	0.9300
C1—C6	1.394 (2)	N24—C25	1.326 (2)
C1—C10	1.533 (2)	C25—H25	0.9300
C2—F2	1.3637 (17)	C11—N11	1.4601 (17)
C2—C3	1.374 (2)	C11—H11A	0.9700
C3—C4	1.382 (2)	C11—H11B	0.9700
C3—H3	0.9300	N11—C15	1.3292 (19)
C4—F4	1.3570 (18)	N11—N12	1.3623 (17)
C4—C5	1.364 (2)	C15—N14	1.3285 (18)
C5—C6	1.388 (2)	C15—H15	0.9300
C5—H5	0.9300	N14—C13	1.355 (2)
C6—H6	0.9300	C13—N12	1.314 (2)
C10—O1	1.4163 (16)	C13—H13	0.9300
C10—C11	1.5296 (19)	O11M—C11M	1.2190 (17)
C10—C21	1.5497 (18)	O12M—C11M	1.3126 (17)
O1—H1	0.89 (2)	O12M—H11	0.88 (2)
C21—N21	1.4558 (19)	C11M—C12M	1.505 (2)
C21—H21A	0.9700	C12M—C13M	1.507 (2)
C21—H21B	0.9700	C12M—H12A	0.9700
N21—C25	1.3311 (19)	C12M—H12B	0.9700
N21—N22	1.3674 (18)	C13M—O22M	1.2063 (18)
N22—C23	1.320 (2)	C13M—O21M	1.3247 (17)
C23—N24	1.358 (2)	O21M—H12	0.88 (3)
			
C2—C1—C6	115.84 (14)	N24—C23—H23	122.4
C2—C1—C10	123.64 (12)	C25—N24—C23	102.34 (13)
C6—C1—C10	120.51 (13)	N24—C25—N21	110.69 (13)
F2—C2—C3	117.17 (13)	N24—C25—H25	124.7
F2—C2—C1	118.65 (13)	N21—C25—H25	124.7
C3—C2—C1	124.18 (14)	N11—C11—C10	112.50 (11)
C2—C3—C4	116.86 (14)	N11—C11—H11A	109.1
C2—C3—H3	121.6	C10—C11—H11A	109.1
C4—C3—H3	121.6	N11—C11—H11B	109.1
F4—C4—C5	119.27 (14)	C10—C11—H11B	109.1
F4—C4—C3	118.27 (14)	H11A—C11—H11B	107.8
C5—C4—C3	122.46 (15)	C15—N11—N12	110.12 (12)
C4—C5—C6	118.58 (14)	C15—N11—C11	130.18 (12)
C4—C5—H5	120.7	N12—N11—C11	119.60 (11)
C6—C5—H5	120.7	N14—C15—N11	109.99 (13)
C5—C6—C1	122.06 (14)	N14—C15—H15	125.0
C5—C6—H6	119.0	N11—C15—H15	125.0
C1—C6—H6	119.0	C15—N14—C13	102.69 (12)
O1—C10—C11	104.54 (11)	N12—C13—N14	115.10 (13)
O1—C10—C1	110.91 (11)	N12—C13—H13	122.5
C11—C10—C1	111.62 (11)	N14—C13—H13	122.5
O1—C10—C21	109.67 (11)	C13—N12—N11	102.10 (12)
C11—C10—C21	109.17 (11)	C11M—O12M—H11	111.3 (14)
C1—C10—C21	110.74 (11)	O11M—C11M—O12M	123.77 (13)
C10—O1—H1	110.4 (13)	O11M—C11M—C12M	123.54 (13)
N21—C21—C10	111.39 (11)	O12M—C11M—C12M	112.67 (12)
N21—C21—H21A	109.3	C11M—C12M—C13M	110.69 (12)
C10—C21—H21A	109.3	C11M—C12M—H12A	109.5
N21—C21—H21B	109.3	C13M—C12M—H12A	109.5
C10—C21—H21B	109.3	C11M—C12M—H12B	109.5
H21A—C21—H21B	108.0	C13M—C12M—H12B	109.5
C25—N21—N22	109.74 (13)	H12A—C12M—H12B	108.1
C25—N21—C21	127.41 (13)	O22M—C13M—O21M	124.14 (14)
N22—N21—C21	122.58 (12)	O22M—C13M—C12M	123.20 (13)
C23—N22—N21	101.97 (12)	O21M—C13M—C12M	112.65 (12)
N22—C23—N24	115.26 (14)	C13M—O21M—H12	112.1 (16)
N22—C23—H23	122.4		

### Hydrogen-bond geometry (Å, º)

**Table:**

*D*—H···*A*	*D*—H	H···*A*	*D*···*A*	*D*—H···*A*
O1—H1···O11*M*	0.89 (2)	1.94 (2)	2.8057 (14)	166.2 (18)
O12*M*—H11···N14^i^	0.88 (2)	1.86 (2)	2.6830 (17)	156 (2)
O21*M*—H12···N24^ii^	0.88 (3)	1.89 (3)	2.7606 (17)	171 (2)
C6—H6···N14^i^	0.93	2.61	3.484 (2)	156
C12*M*—H12*B*···O11*M*^iii^	0.97	2.44	3.3880 (18)	165
C13—H13···O12*M*^iv^	0.93	2.54	3.3144 (19)	141
C25—H25···O11*M*	0.93	2.40	3.2018 (18)	144
C25—H25···O22*M*^iii^	0.93	2.37	3.0032 (19)	125

Symmetry codes: (i) −*x*+1, *y*, −*z*+3/2; (ii) −*x*+1/2, *y*−1/2, *z*; (iii) −*x*+1/2, *y*+1/2, *z*; (iv) *x*+1/2, *y*−1/2, −*z*+3/2.
